# General practice characteristics associated with pay-for-performance in the UK: a systematic review

**DOI:** 10.3399/BJGPO.2024.0174

**Published:** 2025-05-21

**Authors:** Rhatica Srai, David Cromwell, Nicholas Mays, Luisa M Pettigrew

**Affiliations:** 1 Department of Health Services Research and Policy, Faculty of Public Health and Policy, London School of Hygiene & Tropical Medicine, London, UK

**Keywords:** quality assurance, practice organisation, systematic review, general practice, health inequities

## Abstract

**Background:**

The Quality and Outcomes Framework (QOF), a pay-for-performance programme, has been the most widespread quality initiative in NHS general practice since 2004. It has contributed between 25% and 8% of practices’ income during this time, but concerns about its effect on equity have been raised.

**Aim:**

To understand which practice characteristics are associated with QOF performance.

**Design and setting:**

A systematic review was conducted, focusing on NHS general practice in the UK.

**Method:**

MEDLINE, Embase, CINAHL+, Web of Science, and grey literature were searched for studies examining the association between general practice characteristics and QOF performance.

**Results:**

Twenty-two studies, published between 2006 and 2022, exploring the relationship between six population and 15 organisational characteristics and QOF measures were found. Most studies were cross-sectional, of English general practices, and used data from the early years of QOF. A negative association was frequently found between overall QOF performance and socioeconomic deprivation; increasing proportion of registered patients aged >65 years; increasing list size; increasing mean GP age; and Alternative Provider Medical Services contracts. Group practices (versus single-handed), more full-time equivalent (FTE) GPs, and being a training practice were frequently associated with better overall QOF performance. The associations of most other characteristics with performance were inconsistent.

**Conclusion:**

Associations with characteristics both within and outside practices’ control were identified. Pay-for-performance instruments may systematically disadvantage practices serving those at greatest risk of ill-health such as older and more deprived populations. Given the cross-sectional design of many studies and focus on the early years of QOF, more up-to-date evidence is needed to understand if and why these relationships persist.

## How this fits in

The Quality and Outcome Framework (QOF) has been the most widespread service quality initiative in UK NHS general practice over the past 20 years. This systematic review examined studies of the association between QOF performance and general practices’ population and practice characteristics. Associations were identified with characteristics both within and outside practices’ control. Some of these may be exacerbating inequities in health and care. Up-to-date evidence is needed to understand whether and, if so, why these relationships persist as the scope of QOF is reviewed and new pay-for-performance schemes are introduced in general practice in the UK and elsewhere.

## Introduction

The Quality and Outcomes Framework (QOF) was introduced in 2004 to UK NHS general practice as a pay-for-performance incentive scheme. It was viewed as a mechanism to increase government funding into general practice while trying to ensure value for money. The QOF covers a range of clinical and organisational quality indicators that are revised every year.^1–3^ It has been associated with improved recording of, and reduced variation in, incentivised care, but evidence is limited on its impact on health outcomes and health inequalities.^4–17^

QOF scores are publicly reported and in England they inform the Care Quality Commission’s (CQC) regulatory inspections and ratings. The QOF initially contributed up to 25% of practice income, however, it was removed in 2016 in Scotland and its contribution to practice income in England had declined to around 8% by 2022–2023.^18–20^ Despite this, it remains the most widespread quality incentive scheme used in UK general practice over the past 20 years. In 2022–2023, NHS England reported spending £769 million on QOF payments.^19^ Pay-for-performance indicators similar to those in QOF now form part of NHS Primary Care Networks’ (PCN) Investment and Impact Fund (IIF) in England and other pay-for-performance schemes are widespread in the UK general practice under Local Enhanced Services (LES).^21,22^

Various population (for example, location, patient demographics) and organisational characteristics (for example, list size, training practice status) have been found to be associated with general practices’ performance as measured by the QOF at different time points, across different QOF measures and geographical areas. However, this evidence has not been reviewed as a whole. This study therefore systematically reviews evidence, using national-level data from the four countries in the UK, to understand which general practice characteristics have been studied and their associations with QOF.

## Method

The review is reported in accordance with Preferred Reporting Items for Systematic Reviews and Meta-Analyses (PRISMA) and Synthesis Without Meta-analysis (SWiM) in Systematic Review guidelines.^23,24^ The protocol was registered with PROSPERO.^25^

### Search strategy

MEDLINE, Embase, CINAHL+, and Web of Science databases were searched using terms related to (i) QOF and (ii) statistical measures of association (Supplementary Box 1) up to January 2022. The reference lists of selected articles were searched for additional studies and Google was used to search for grey literature using keywords from the search strategy.

### Inclusion and exclusion criteria

Studies were included if they examined whether QOF performance was associated with any population or organisational general practice characteristics using national datasets from England, Scotland, Wales and/or Northern Ireland. The review excluded studies that used QOF or other performance measures as explanatory variables in their models, or used QOF exception reporting rates (when patients are excluded from the eligible QOF population for a justified reason)^26^ as the outcome variable.

### Study screening, selection, data extraction, and quality assessment

RS and LP independently screened and selected the studies. Both extracted data and quality-assessed the studies with disagreement resolved by discussion between the authors. Quality was assessed using the modified Newcastle-Ottawa Scale for cross-sectional studies and Critical Appraisal Skills Programme (CASP) tool for cohort studies.^27,28^ Data were extracted on the population, study design, year(s) of data used, exclusion criteria, explanatory and outcome variable(s), adjustment for confounding, direction of associations and their statistical significance. Associations were taken from the final statistical model(s) published, where available, including supplementary material.

### Categorising characteristics and synthesis

#### Explanatory variables

Where possible, related explanatory variables were grouped. For example, various measures of deprivation were combined into one group. Variables were left in subcategories if they could not be combined in a logical way owing to a lack of overlap, for example, some patient age groupings.

#### Outcome variables

The protocol was revised to group QOF outcome variables into three categories, rather than one, owing to the range used in studies.^25^ The categories were as follows: (i) ‘overall’ where the total QOF or whole domain(s) (for example, ‘clinical’ domain) scores were used; (ii) ‘subdomain’ where one or more disease or condition specific QOF subdomain(s) scores were used (for example, asthma, diabetes mellitus, mental health; if only the register indicator, which captures reported prevalence, was omitted, then this was still considered as a full subdomain); and (iii) ‘subgroup’ where a group of indicators had been selected by the study authors (for example, influenza immunisation, blood pressure, cholesterol). Associations with the percentage of QOF points achieved, with (‘reported achievement’) and without (‘population achievement’) exception reporting were counted separately. Studies were grouped by the time period of the QOF figures into early, mid, and recent periods to identify time trends. If multiple years of data were reported separately, each year was counted as a separate analysis as QOF indicators, target thresholds, and associated payments changed over time.

#### Synthesis

Heterogeneity between studies precluded synthesis beyond capturing the direction and strength of association. Associations were classed as ‘positive’, ‘negative’, or ‘no association’. Associations that were not statistically significant (*P*>0.05) were classified as ‘no association’. Associations reported after adjusted for cofounding factors scored one point; unadjusted associations, where they were the final results, were awarded half a point. Points per association were added within and across all studies to give the direction and consistency of association. If all associations were in the same direction, we considered this a ‘consistent’ association. If ≥60% of associations were the same direction, this was considered to be a ‘relatively consistent’ association. If <60% of the associations were in the same direction, this was considered to be an ‘inconsistent association’.

## Results

**Figure 1.**
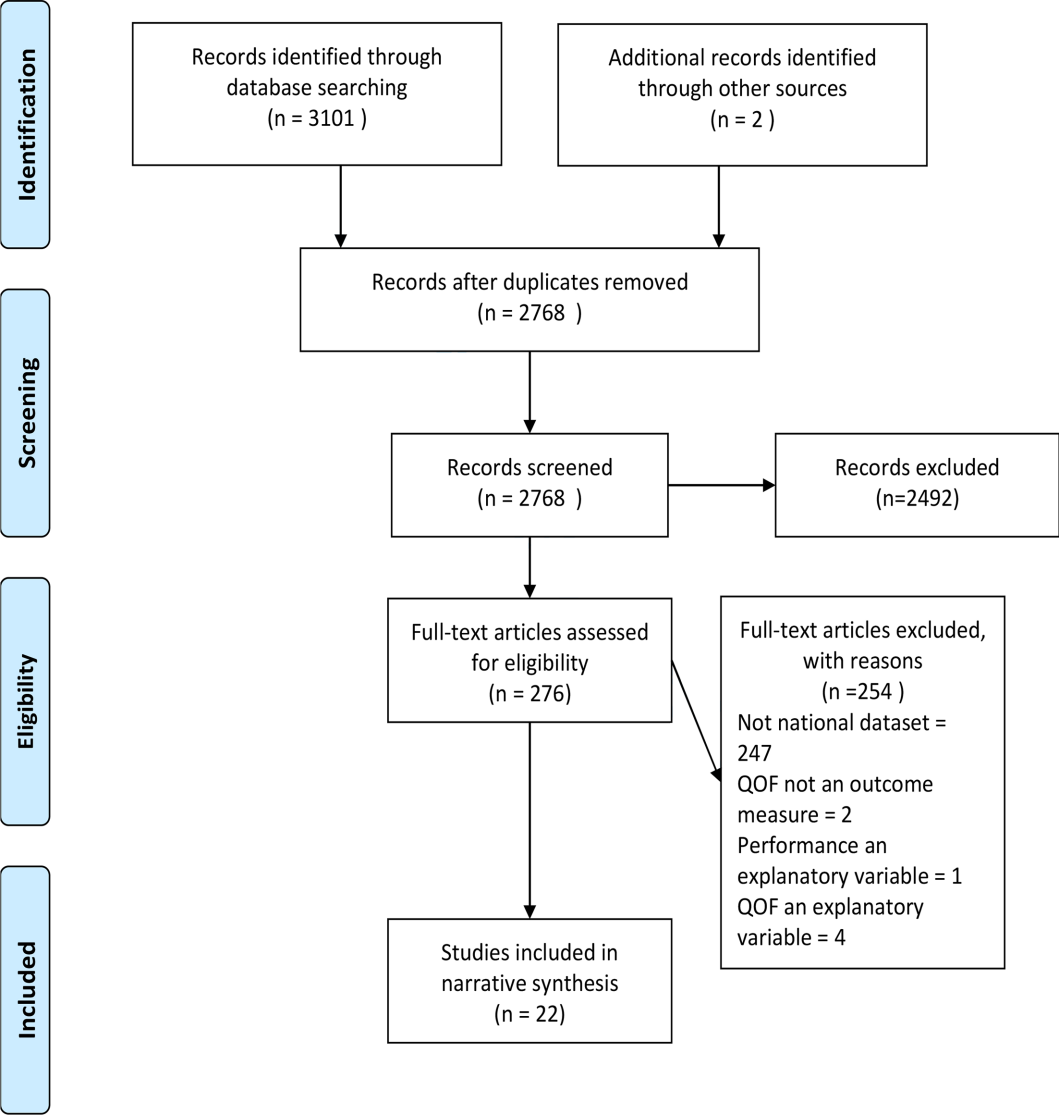
Preferred Reporting Items for Systematic Reviews and Meta-Analyses (PRISMA) flowchart of study selection. QOF = Quality and Outcomes Framework

Search strategy results are summarised in Figure 1. Twenty-two studies published between 2006 and 2022 were included: 15 cross-sectional and seven cohort studies. Study characteristics are detailed in Supplementary Tables S1 and S2.

### Study locations and time periods

Sixteen studies were based in England,^14,29–43^ three in England and Scotland (one of which examined the countries separately),^44–46^ and three in Scotland.^47–49^ We found no studies from Northern Ireland or Wales. Most studies excluded practices (i) with <1000 registered patients owing to their atypical nature, (ii) with missing data, or (iii) that were not consistently within merged datasets. Most studies reported including more than 90% of practices in the UK country studied (Supplementary Tables S1 and S2). A description of the typical characteristics of excluded smaller practices can be found elsewhere.^7^

Studies used QOF data from 2004/2005 to 2016/2017, but 14 studies examined the first two years of QOF implementation. Longitudinal studies varied from 2–5 years, two presented the years separately,^14,38^ and the remainder reported average values over the study period^31,34,37,41,49^ (Supplementary Tables S1 and S2).

### Methodological quality of studies

Fourteen studies were rated as being of high methodological rigour,^14,29–31,33,35–42,49^ four were rated as good,^34,43–45^ and four as satisfactory^32,46–48^ (Supplementary Tables S3 and S4).

### Type and frequency of explanatory characteristics studied

Twenty-one explanatory variables — six population and 15 organisational characteristics — were included in studies. Which, how often, and for what purpose these were explored varied. For example, computer system was only studied once, while deprivation was used in 17 studies in various formats. Fifteen studies included multiple explanatory characteristics in their regression model(s) and adjusted for confounding. Two adjusted for confounding in a very limited way (that is, only included two explanatory variables),^43,45^ five did not adjust at all (for example, reported univariate analysis)^32,44,46–48^ (Supplementary material Tables S3 and S4).

### Type and frequency of QOF outcomes studied

Most studies examined associations with more than one QOF measure. Overall measures of QOF achievement were used in 12 studies, with the ‘clinical domain’ being the most studied;^14,29–32,35,37,38,41–43,49^ subdomains were used in eight studies;^33,34,38–40,44–46^ and subgroups in twelve.^30,34,36,38–41,44,46–49^

### Consistency of associations

**Figure 2.**
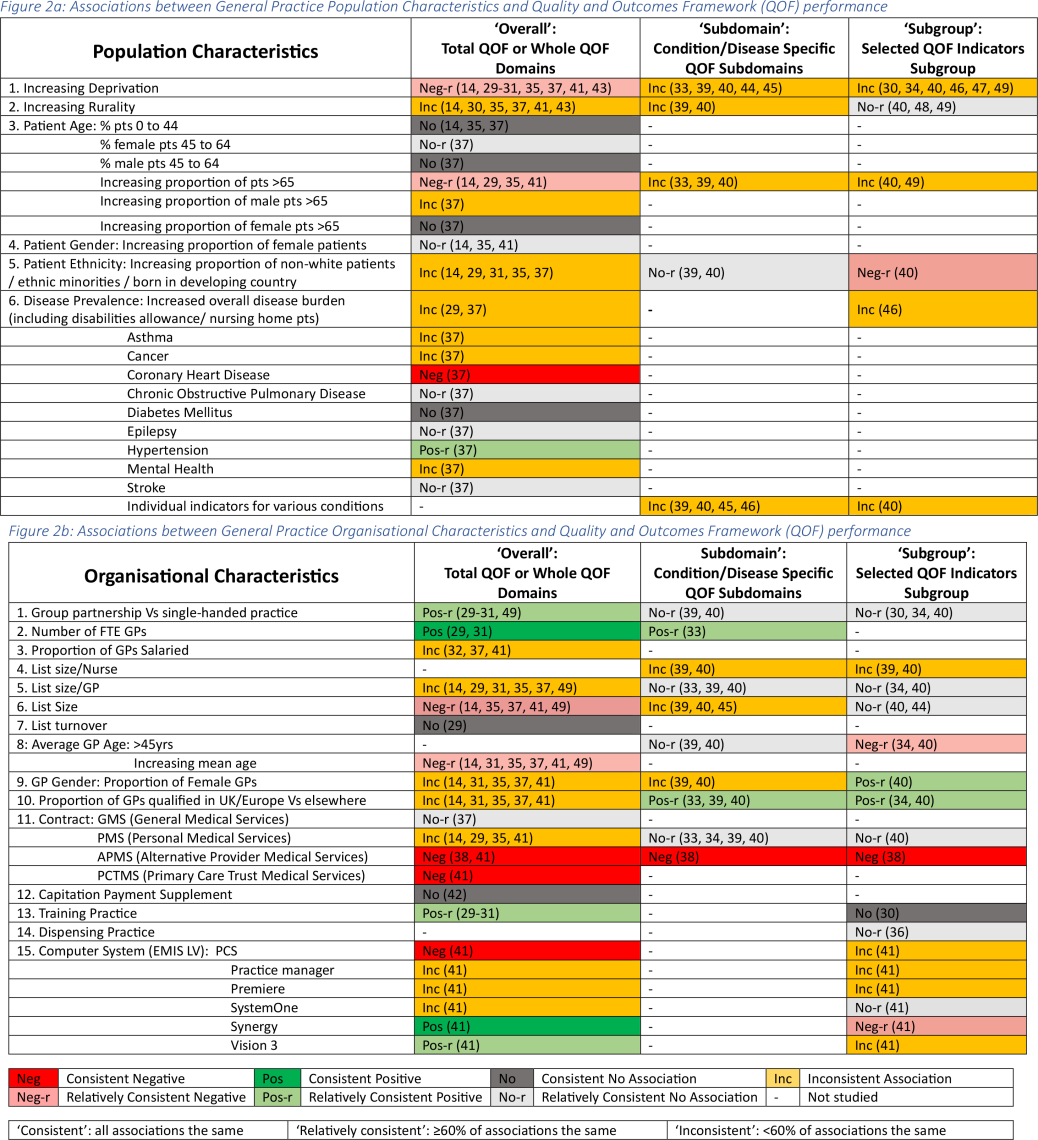
a: Associations between general practice population characteristics and Quality and Outcomes Framework (QOF) performance. b: Associations between general practice organisational characteristics and QOF performance. (Numbers in brackets represent the study citations). FTE = full-time equivalent. PCS = name of software.

Associations that were examined in two or more studies and that showed consistent or relatively consistent positive or negative associations for the ‘overall’ QOF performance category, as well as the most frequently studied explanatory variables are discussed below. All explanatory variables with their direction and consistency of association with QOF performance are presented in Figure 2a&b with the relevant citations. Full data extraction tables are available from the authors.

#### Population characteristics

Deprivation was the most studied characteristic, showing a relatively consistent negative association with the overall QOF performance,^14,29–31,35,37,41,43^ but an inconsistent relationship with specific clinical subdomains^33,39,40,44,45^ and indicator subgroups.^30,34,40,46,47,49^ Rurality was the second most frequently studied explanatory variable. This showed an inconsistent association with overall QOF performance^14,30,35,37,41,43^ and subdomains,^39,40^ and relatively consistently no association with indicator subgroups.^40,48,49^

Having a higher proportion of patients aged >65 years was frequently studied and showed a relatively consistent negative association with overall QOF achievement,^14,29,35,41^ but an inconsistent relationship with subdomains and indicator subgroups.^33,39,40,49^ Patient ethnicity was also frequently studied and showed an inconsistent association with overall achievements^14,29,31,35,37^ and relatively consistently no associations with subdomain achievements.^39,40^ In one study examining subgroup indicators, there was a relatively consistent negative association between the percentage of patients from an ethnic minority and QOF performance, driven by indicators related to diabetes and epilepsy.^40^

#### Organisational characteristics

Group practices (versus single-handed practices) were relatively consistently associated with better overall achievement;^29–31,49^ however, this association did not hold when examining clinical subdomains and indicator subgroups,^30,34,39,40^ which had relatively consistent no association. Similarly, higher numbers of full-time equivalent (FTE) GPs were consistently associated with better overall performance.^29,31^ This association was also present in clinical subdomains, although to a lesser degree.^33^ In contrast, there was a relatively consistent negative association with list size^14,35,37,41,49^ and an inconsistent relationship between list size per GP and overall performance.^14,29,31,35,37,49^

Increasing mean GP age was relatively consistently associated with poorer overall performance,^14,31,35,37,41,49^ notably for GPs aged >45 years in indicator subgroups related to diabetes and stroke.^40^ Seven studies examined the association with GP gender ^14,31,35,37,39–41^ and reported inconsistent associations with achievement, except in one study of subgroup indicators where female GPs showed a relatively consistent positive association with QOF achievement, driven by diabetes and epilepsy indicators.^40^ The proportion of GPs qualified in the UK or the rest of Europe (versus elsewhere) was frequently studied. While there was an inconsistent association with overall QOF measures,^14,31,35,37,41^ a higher proportion of UK or rest of Europe qualified GPs was relatively consistently associated with higher achievement in clinical subdomains and indicator subgroups driven by chronic obstructive pulmonary disease (COPD), coronary heart disease, diabetes, epilepsy, hypertension, hypothyroid and stroke indicators.^33,34,39,40^

The relationship with different NHS contract types was also frequently analysed. Two studies examined Alternative Provider Medical Services (APMS) contracts and both showed consistently negative associations across all QOF groupings.^38,41^ Training practice status was relatively consistently associated with better overall performance measures in three studies.^29–31^

#### Trends in associations

We did not identify any differences in patterns of association comparing QOF measures with or without exception reporting, nor comparing specific clinical subdomain or indicator subgroup explanatory variables. We also did not find any trends in associations over time or in the three Scottish studies compared with those in England, although ability to do so was limited owing to the lack of studies in more recent years and from outside England.

## Discussion

### Summary

Twenty-two studies were found exploring the relationship between 21 population and organisational general practice characteristics and QOF performance. Most studies used data from the early years of QOF in England. An association was frequently identified between poorer overall QOF performance and higher deprivation; increasing proportion of patients aged >65 years; increasing list size; increasing mean GP age; and having an APMS contract. A positive association with overall QOF performance was frequently seen with group practices (versus single-handed); total FTE GPs; and training practices. The proportion of GPs whose primary medical qualification was from the UK or Europe (versus elsewhere) showed a relatively consistent positive association with better performance across QOF subdomains and subgroup indicators, but an inconsistent association with overall performance. Inconsistent associations were found with most other characteristics.

### Strengths and limitations

We only included studies using national datasets as QOF was a national policy; however, an opportunity exists to examine sub-national studies. Variables that had multiple definitions were grouped to make sense of the findings but in the process lost granularity. Most studies used cross-sectional data from the first few years of QOF; however, practice characteristics, contextual factors, and QOF itself have changed over time.^50^ The use of vote-counting across heterogenous studies has limitations, including not being able to comment on the magnitude of associations and the risk of subjective interpretation.^51^ Counting only statistically significant associations, giving less weighting to studies that did not adjust for confounding, and setting a 60% rather than a 50% cut-off to define the consistency of associations reduced the risk of overestimating the presence of these, although it may have resulted in an underestimation.^24,51^ Importantly, association does not mean causality; however, it does signal areas that merit further attention, in particular, where associations appear more consistently and are plausible causally.^52^

### Comparison with existing literature

Studies have shown that inequalities in performance related to levels of deprivation diminished during the early years of QOF performance.^14,15,53^ However, the association persisted in studies using later data and is seen in sub-national level studies.^53,54^ The association between poorer performance and proportion of patients aged >65 years contrasts with findings of a recent longitudinal study suggesting practices with a higher proportion of patients aged >65 years perform better on QOF; this difference may be owing to methodological differences adjusting for confounding variables.^53^ Associations between poorer performance, indices of deprivation, and older populations suggest that the socioeconomic determinants of health and the ‘inverse care law’ may be at play in determining practices’ ability to perform on QOF measures.^55–59^

The mixed picture of associations with the number of FTE GPs, list size and patients or FTE GP may reflect differences in methods and data used. However, it mirrors mixed findings in the wider literature regarding the relationship between list size and clinical quality of care, suggesting variables other than organisational size drive performance and that the relationship may not be linear.^37,60–62^

The association between older GPs and poorer performance on QOF may seem counterintuitive and could indicate intentional disengagement from QOF; for example, to prioritise other dimensions of quality, rather than an inability to deliver. However, a 2005 systematic review also identified a negative relationship between clinical experience and measured quality of health care.^63^ New evidence in this area would be helpful as the volume of information and mechanisms for clinicians to keep up to date have changed considerably.

Around 25% of GPs working in England qualified outside the UK.^50,64^ Their contributions in, typically, more challenging and socioeconomically deprived areas has often been under-recognised and, while hypothesised, evidence that international medical graduates deliver poorer quality care has been limited to date.^65–67^ However, an association remained after controlling for socioeconomic deprivation between better performance in some aspects of QOF and practices with a higher proportion of UK/European-qualified GPs.

APMS service contracts are time-limited, they account for a small (4% in England) and diminishing proportion of general practice contracts and are often used to enable the contracting of incorporated limited companies.^19,68,69^ Greaves *et al* identified that practices with APMS contracts are usually smaller, serving younger, more diverse and deprived populations, but despite adjustment still found an association with poor performance.^38^ Commercial interests and the use of APMS contracts when failing practices have been put out to tender have been hypothesised as reasons for poorer performance.^38^ In contrast, the proportion of training practices is increasing,^50^ and these have been associated with better clinical quality as well as patient satisfaction owing to their greater focus on education and clinical governance.^64,70–73^

### Implications for research and practice

This review identifies commonly used measurable population and organisational general practice characteristics. It identifies that up-to-date research into practice characteristics associated with QOF is needed. Synthesising existing evidence at sub-national level and comparing associations with other quality measures, notably the General Practice Patient Survey (GPPS), CQC ratings, and other pay-for-performance schemes, such as LESs and the IIF, would inform policy regarding general practice funding and its organisational structure. Given the evolving structure of general practice, other explanatory characteristics could be examined including the ratio of non-GP:GP FTE roles per 1000 patients; or the use of different digital solutions to drive quality, noting that Kontopantelis *et al* found that the choice of clinical computing system was the strongest predictor of QOF performance in their model.^41^

Pay-for-performance schemes, while they have the potential to help address inequities in health and care, if not carefully designed, they may exacerbate these inequities.^16,74,75^ Practices in socioeconomically deprived neighbourhoods need adequately adjusted capitated funding and support to address the social determinants of health, as well as to care for older populations who have greater multimorbidity.^76–79^

Caution is needed with current policy driving the formation of larger general practice organisations and task shifting to allied healthcare professionals, as its relationship with quality and cost-effectiveness is unclear.^37,50,60,61,80,81^ The associations between increasing mean GP age and being qualified outside the UK or Europe requires further investigation, but may suggest the need for careful workforce planning and additional support for certain GP cohorts to engage with continuous professional development.

Inconsistencies in associations with other practice characteristics and QOF may reflect methodological differences, such as the QOF performance measure used, year of study, and degree of adjustment for confounding. They may also be due to to non-linear relationships. Importantly, inconsistencies in associations highlight the complexity of quality as a concept, and the limitations of quantifiable characteristics being able to explain variation.^9,82,83^

In conclusion, relatively consistent associations with QOF performance and characteristics that are within practices’ control and those that are not were found. Up-to-date evidence is needed to understand if and why these relationships persist as they may be exacerbating inequities in health and care that need to be addressed.

## Trial registration number

PROSPERO (CRD42021225146)

## Data

All data used in this review were taken directly from the articles cited, which are publicly available.
